# Hypertrophic Pachymeningitis: An Unusual Cause of Headache

**DOI:** 10.7759/cureus.53576

**Published:** 2024-02-04

**Authors:** Joud Enabi, Muhammad Waqar Sharif, Raksha Venkatesan, Hema Kondakindi, Maida Faheem

**Affiliations:** 1 Internal Medicine, Texas Tech University Health Sciences Center, Odessa, USA; 2 Internal Medicine, Midland Memorial Hospital, Midland, USA

**Keywords:** chronic headache, granulomatosis with polyangiitis, meningeal inflammation, anca-associated vasculitis, hypertrophic pachymeningitis

## Abstract

Hypertrophic pachymeningitis (HP) is a rare condition characterized by inflammation and thickening of the dura mater. It can be idiopathic or secondary to various causes, including infections, tumors, or systemic inflammatory diseases. Diagnosis is challenging due to its rarity and the overlap of symptoms with other conditions.

We present the case of a 42-year-old Hispanic woman with diabetes mellitus type 2 and end-stage kidney disease who presented with chest pain, dry cough, mild dyspnea, and chronic occipital headaches. Physical examination revealed cranial VI nerve palsy. Imaging showed pulmonary cavitary lesions and mediastinal lymphadenopathy. Elevated inflammatory markers and positive autoimmune tests, including rheumatoid factor and antineutrophil cytoplasmic antibody (ANCA), led to further investigation. Brain imaging revealed dural thickening, confirming HP. The patient's medical history revealed double ANCA positivity and a lung biopsy confirmed granulomatous pneumonitis. A diagnosis of ANCA-associated vasculitis (granulomatosis with polyangiitis (GPA)) was established, and treatment with rituximab and high-dose corticosteroids led to symptom improvement.

GPA rarely involves meningeal inflammation, but severe and persistent headaches are common early symptoms. Inflammatory markers are often elevated, and around two-thirds of HP cases related to GPA have positive serum ANCA. MRI is the primary diagnostic tool, with characteristic findings of dural thickening and contrast enhancement. This case highlights HP as a rare cause of chronic headaches and the importance of a comprehensive medical history in diagnosis. Early recognition and treatment are crucial for improving outcomes in GPA-related HP.

## Introduction

Hypertrophic pachymeningitis (HP) is a condition of localized or diffuse inflammation and thickening of the dura mater [1]. This condition may be based on etiology as either “primary,” also known as “idiopathic,” or “secondary” [2] to infectious diseases, nearby tumors, or systemic inflammatory diseases [1]. It may also be based on the location of the lesion as either cranial or spinal pachymeningitis [1]. The most common presentation is headache, which could be a diagnostic challenge due to the broad differentials of headaches and the rare incidence of HP [3]. Cranial nerve defects could also be a clinical manifestation, with cranial nerve II defects being the most common, followed by cranial nerves VI, VIII, III, IV, and V. Additional clinical features include psychiatric disorder [4,5], ataxia, and seizures. Other symptoms include numbness, bladder and rectal dysfunction, back pain, and nerve root or spinal cord compression. This condition can be seen as gadolinium enhancement on MRI. The diagnosis is based on biopsy and imaging findings [6].

The prevalence of HP is low, thus making it challenging to analyze the disease in detail and understand a holistic approach to its progress. This rare disease has a prevalence reported as 0.949/100,000 persons, and of those affected, the majority are known to be of Caucasian background. Idiopathic HP contributes to nearly half of the prevalence cases studied [7]. This case report analyzes a rare case of ANCA-related HP.

This article was previously presented as a meeting abstract at the Society of General Internal Medicine on March 22, 2023.

## Case presentation

**Figure 1:**
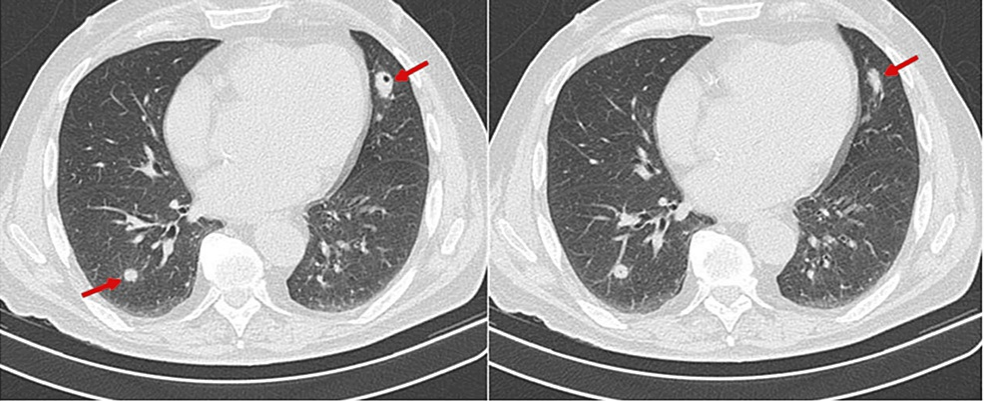
(Left, right) CT scan of chest Red arrows pointing toward multiple lung nodules of various sizes in bilateral lung fields

**Figure 2:**
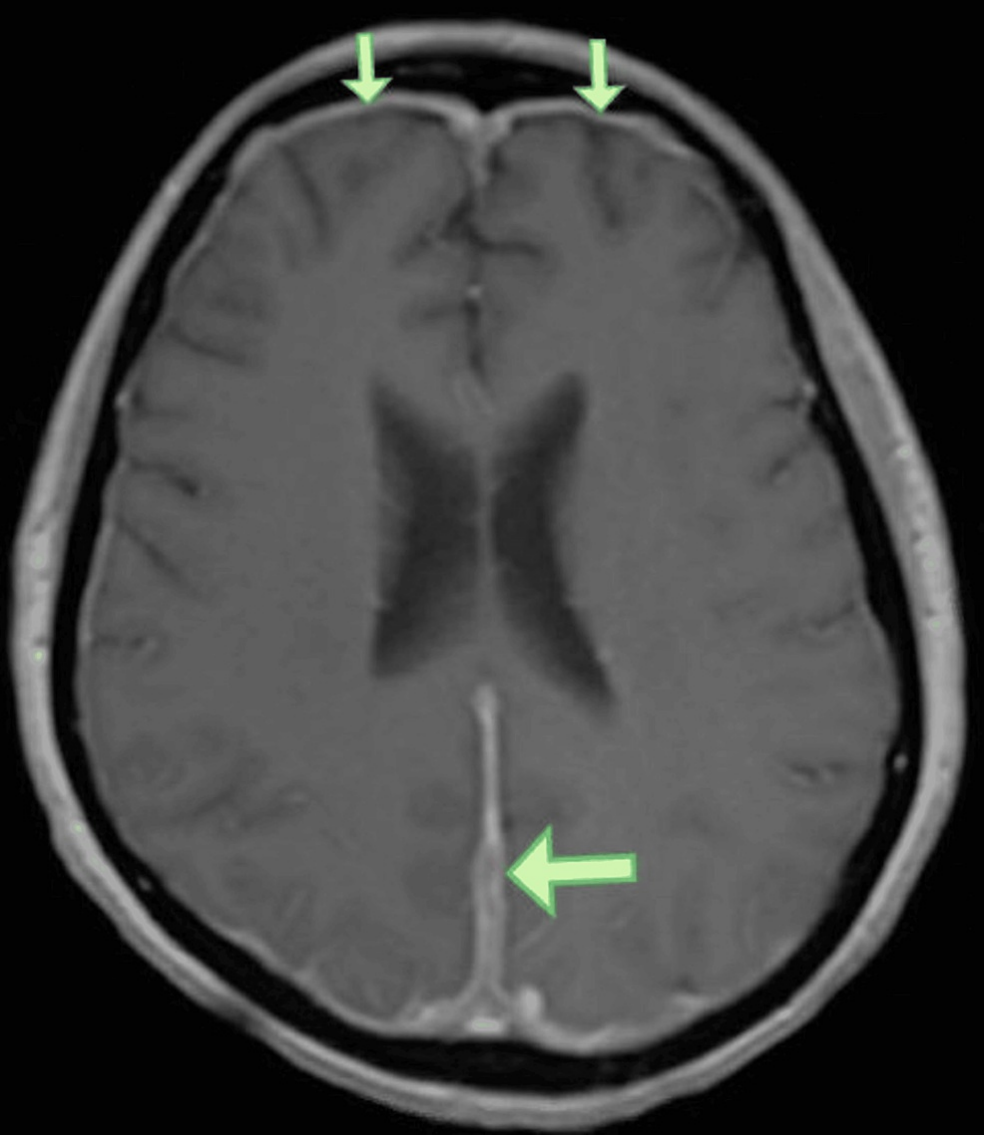
MRI of brain w/wo gadolinium contrast, T1 sagittal view Green arrows pointing toward the contrast enhancement of the meninges.

**Figure 3:**
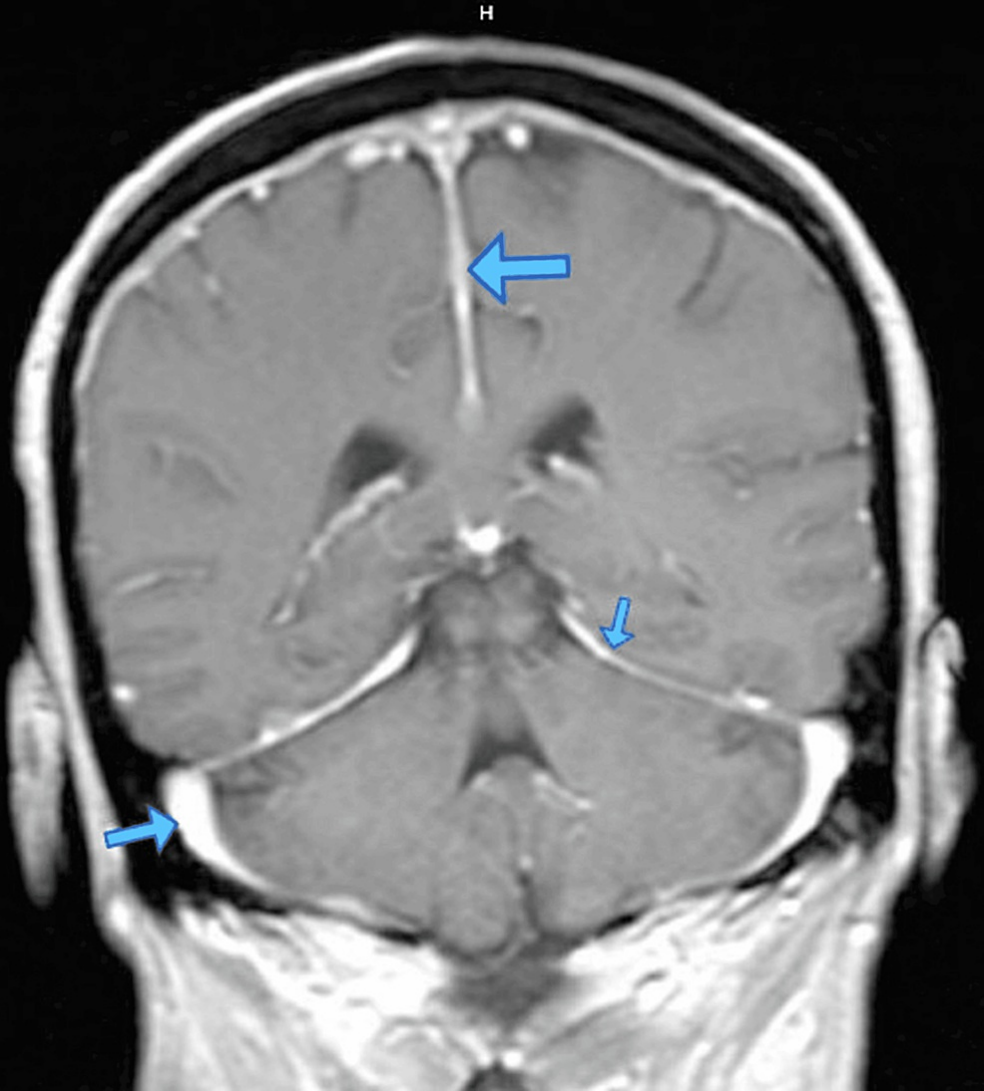
MRI of brain w/wo gadolinium contrast, T1 coronal view Blue arrows pointing toward enhancement of the meninges

A 42-year-old Hispanic female with a past medical history of type 2 diabetes mellitus, morbid obesity, and end-stage renal disease presented to the Emergency Department initially for new-onset pleuritic chest pain. She described the pain as severe, non-radiating, mainly in the midsternal region, associated with dry cough and mild dyspnea. In addition, she has been complaining of chronic occipital headaches for the past few months. Physical exam was notable for left cranial VI nerve palsy and bilateral scattered inspiratory crackles. Chest CT scan with contrast showed bilateral multilobar cavitating pulmonary nodular lesions measuring 2.7 cm and smaller with a predilection for the juxta pleural structures (Figures 1, 2). Low-grade mediastinal lymphadenopathy was demonstrated with small bilateral pleural effusions. The diagnostic differentials per CT included septic pulmonary emboli and cavitating hematogenous metastases, amongst others. Broad-spectrum antibiotics were started. In view of the chest pain, troponin was also checked, which were 0.22ng/mL, 0.35ng/mL, and 0.5ng/mL, and trended down. EKG was not suggestive of any T- or ST-wave changes. The cardiologist deemed it demand ischemia. Due to a question of metastasis, CT of the abdomen and pelvis, and CT of the Brain were done to screen for malignancy. CT abdomen was unremarkable, and head CT demonstrated diffuse thickening of the cerebral falx extending to the right tentorial leaf near the tentorial incisura, which was confirmed on brain MRI with contrast (Figures 2, 3). A biopsy of the right lung showed Necrotizing granulomatous pneumonitis and was negative for malignancy, AFB stain, and fungal infections. Inflammatory markers and autoimmune disease results showed ESR > 100, elevated CRP, negative RPR, and HIV, positive Rheumatoid Factor (RA titer - 64), Antineutrophil cytoplasmic antibodies (ANCAs) level < 1:20, no pattern was detected, more labs are given in Tables 1, 2. MPO titer - 0, and PR3 antibody - 3. Analysis of cerebrospinal fluid was unremarkable except for elevated protein of 53 and negative for bacterial and fungal elements. Additional history from her former nephrologist revealed that ESRD was associated with double ANCA positive. At this point, a diagnosis of ANCA-associated vasculitis was established. She was started on Rituximab (375 mg/m² IV weekly for four weeks) and Solu-Medrol 500 mg IV daily for three days, followed by prednisone 50 mg p.o. daily with a gradual taper of prednisone 5 mg down every week. Her presenting symptoms improved after three days of IV steroid treatment. This case illustrates HP as a rare etiology of chronic headaches and the value of a complete and thorough history.

**Table 1:** Cerebral spinal fluid analysis

CSF Hematology	
Appearance CSF	Hazy
Color	Colorless
WBC	14
RBC	1,626
Segmented Neutrophils	66
Lymphocyte	19
Monocyte	5
Cerebrospinal Fluid (CSF) Chemistry	
Glucose	40
Protein	53

**Table 2:** Autoimmune workup

Immunology/Serology		
RPR (rapid plasma reagin)		Non-Reactive
Autoimmune Disease/testing		
Rheumatoid Factor	Positive (A)	
Rheumatoid Arthritis titer	64.0	
Serine Proteinase 3, IgG	12	
Myeloperoxidase (MPO) Antibody IgG	0	
Antineutrophil Cytoplasmic Antibodies Pattern	Non-Detected	
Antineutrophil Cytoplasmic Antibodies Titer	<1:20	

## Discussion

HP is an uncommon neurological disorder, and there has been no extensive epidemiological study conducted on a large scale. Its reported incidence rate is approximately 0.949 per 100,000 individuals [3,7]. Initial symptoms of this condition tend to be mild and nonspecific, making them easily overlooked and misdiagnosed. Delays in accurate diagnosis and treatment can result in the involvement of multiple sites within the nervous system, leading to recurrence and unfavorable prognosis [8]. The etiology of HP is multifaceted and varied, encompassing factors such as autoimmune diseases (e.g., rheumatoid arthritis), infections (e.g., tuberculosis, syphilis), systemic conditions (e.g., IgG4-related diseases), neoplasms (e.g., lymphoma), and other confounding elements. Instances of HP without a clear etiology are termed idiopathic HP (IHP) [9-11]. Predominantly, headache constitutes the principal clinical manifestation [12,13], and the second most frequent presentation involves cranial nerve deficits, with the optic nerve being the most commonly affected, followed by cranial nerves III, IV, V, VI, and VIII [14]. Additional clinical features encompass epileptic seizures, auditory impairment, sensory loss, abnormal movements, lack of muscular coordination, and positive pathological reflexes [13]. Myelin oligodendrocyte glycoprotein antibody-associated disease (MOGAD) is a rare autoimmune disorder triggered by MOG antibodies, which induce demyelination within the central nervous system, predominantly impacting the optic nerve, meninges, spinal cord, and brainstem [15,16]. The exact pathogenic mechanisms underpinning IHP linked to MOG antibodies remain unclear. The clinical manifestation of MOG antibody-related IHP lacks differentiation from other forms of HP and results from mechanical compression of vascular or nerve structures, culminating in functional deficits [11]. Given the limited specificity of early clinical symptoms associated with HP, employing multiple diagnostic procedures plays a pivotal role in identifying the underlying cause. Prior studies have verified elevated levels of inflammatory markers (high-sensitivity C-reactive protein and erythrocyte sedimentation rate). Approximately 70% of patients exhibit intracranial hypertension alongside elevated leukocyte and protein levels within cerebrospinal fluid [12,13,16]. These patients typically manifest the aforementioned symptoms. Magnetic resonance contrast-enhanced imaging serves as a vital supplementary diagnostic tool, primarily for assessing the location, extent, and enhanced patterns of abnormal dura mater [17]. Tissue biopsy, the gold standard for HP diagnosis, reveals fibrosis of connective tissue and chronic inflammation, characterized by the infiltration of lymphocytes, plasma cells, and/or epithelioid histiocytes [12,13,16,17]. Unfortunately, a biopsy was not conducted in this particular case. Nevertheless, considering the clinical presentation and examination outcomes, the condition was ultimately attributed to immune-related IHP provoked by MOG antibodies.

Treatment strategies for HP hinge on addressing the underlying cause. In cases with confirmed infection sources (e.g., bacterial, fungal, tuberculous, or viral etiologies), etiological treatment takes precedence [16]. For cases of IHP after excluding secondary factors, corticosteroids are the primary therapeutic approach. Steroid therapy effectively mitigates IHP symptoms [13]. A study in Japan demonstrated that 87.2% of 94 patients treated with corticosteroids experienced significant symptom alleviation. For non-responsive patients, combining immunosuppressive agents led to symptom improvement in approximately 92.6% of cases [18,19]. However, refractory instances may necessitate surgical intervention. In the discussed patient, clinical symptoms improved following a year-long course of corticosteroid shock therapy, highlighting the continued efficacy of corticosteroids. Nevertheless, recurrence is a considerable concern in HP treatment. Reports indicate that around 50% of HP patients experience relapse post-treatment, occurring anywhere from one week to several years after initial therapy [20].

## Conclusions

In conclusion, HP is an uncommon neurological disorder that causes inflammation and thickening of the dura mater. The etiology of HP includes autoimmune diseases, infections, systemic causes like IgG4-related disease, neoplasms, or idiopathic diseases like MOGAD. The most common presentation is headache, followed by cranial nerve involvement. The most common lab findings are elevated ESR CRP. MRI of the brain with contrast is the imaging of choice. Tissue biopsy is the gold standard for diagnosing HP. Treatment includes addressing the underlying cause. If the cause is idiopathic, glucocorticoids should be started. Physicians should be aware of this rare disease, as early diagnosis and timely intervention are pivotal determinants of HP prognosis. Due to the high recurrence rate, initiating immunotherapy promptly as needed is advised. Regular MRI evaluations serve as a means to assess nervous system damage and therapeutic outcomes in patients.
